# The Impact of Early Nutrition Following Peroral Endoscopic Myotomy in Achalasia: A Tertiary Center Experience

**DOI:** 10.5152/tjg.2024.24053

**Published:** 2024-03-01

**Authors:** Abdullah Murat Buyruk, Çağdaş Erdoğan, Fatih Tekin

**Affiliations:** 1 Department of Gastroenterology, Ege University School of Medicine, İzmir, Turkey; 2 Department of Gastroenterology, University of Health Sciences, Ankara Etlik City Hospital, Ankara, Turkey

**Keywords:** Peroral endoscopic myotomy, achalasia, enteral feeding

## Abstract

**Background/Aims:**

This retrospective single-center study aimed to assess the safety of early feeding in patients who met certain criteria following peroral endoscopic myotomy (POEM).

**Materials and Methods:**

Data from 100 patients who underwent POEM at our center between January and December 2022 were collected. Early feeding was defined as the introduction of clear liquid foods at 4 hours post procedure. At 4 and 24 hours, the pain was rated using the visual analog scale (VAS) in all patients. Patients without intraoperative complications (pneumoperitoneum requiring needle drainage, severe arterial bleeding requiring the use of hemostatic forceps, severe mucosal injury) and severe pain (VAS score > 6) and nausea-vomiting at the fourth postoperative hour were given the early feeding approach. In patients who did not meet these requirements, enteral feeding was initiated after 24 hours (late feeding).

**Results:**

Among the 100 patients, 50 patients were categorized early feeding. No patients had a control esophagogram. In the early and late enteral feeding groups, VAS scores were 4 (0-6) and 6 (1-8) (*P* < .001) at 4 hours and 1 (0-3) and 1 (0-6) (*P* = .043) at 24 hours, respectively. No severe complications were developed after early feeding. The median hospital stay in the early feeding group was 1 (1-3) day. There was no emergency readmission in any of early feeding patients.

**Conclusion:**

Our study showed early feeding following POEM can be begun in achalasia patients who do not have intraoperative complications, severe pain, or nausea/vomiting.

**Main Points**

- The time of starting to feed after peroral endoscopic myotomy (POEM) procedure remains unknown.
- Early feeding following POEM can be started in achalasia patients who meet certain criteria.
- Early feeding did not cause any post-POEM serious complications.

## Introduction

Achalasia is a rare esophageal disorder affecting the ability of the lower esophageal sphincter (LES) to relax and allow food to pass into the stomach.^1^ The treatment for achalasia typically involves procedures aiming at relaxing the LES and improving the food movement through the esophagus.^2^ This may include medications to help relax the esophageal muscles, pneumatic balloon dilation (PBD) to widen the LES, or surgical procedures such as laparoscopic Heller myotomy (LHM) or peroral endoscopic myotomy (POEM).^3^

Peroral endoscopic myotomy has emerged as a promising treatment option for achalasia, offering patients a minimally invasive alternative to traditional surgical techniques. Several studies demonstrated the safety and efficacy of POEM for the treatment of achalasia, with long-term follow-up indicating sustained improved symptoms and quality of life.^4-8^ A meta-analysis of 21 studies involving 1646 patients found that POEM had success and complication rates of 92.4% and 9.5%, respectively, with the severity of most complications being mild or moderate.^8^ The majority of studies in the last 10 years have demonstrated the usefulness and dependability of the POEM approach, which was originally used by Inoue et al.^9^ in 2008. The POEM approach, which has success rates comparable to surgical treatment and was even the first suggested treatment method for type 3 achalasia, is still available in a limited number of centers nowadays.^10^

We have been observing developments in the postoperative follow-up of patients with great interest in recent days, rather than technical developments in the POEM method. The best timing for initiation of enteral feeding or for discharge after POEM is still unknown. The consideration of initiating feeding on the same postoperative day and possibly discharging patients on the same day after the POEM procedure has gained prominence during the COVID-19 pandemic.^11^ However, only a few studies reported that the POEM procedure may be performed with same-day discharge following early feeding. Four studies reported success rates of 48%-82.4% after POEM with same-day discharge following early feeding.^11-16^ In conclusion, the timing of the start of enteral feeding following POEM remains unknown.

This study aimed to assess the safety of early feeding in patients who met certain criteria following POEM.

## Materials and Methods

### Patients

This single-center retrospective study screened data from 121 individuals who underwent POEM at Ege University Faculty of Medicine, Department of Gastroenterology, between January and December 2022. Early feeding was defined as the introduction of clear liquid food 4 hours post procedure. The primary physician who conducted the procedure scored pain using the visual analog scale (VAS) at 4 and 24 hours post procedure in all patients. Patients were graded as 0, no pain; 1-3, mild; 4-6, moderate; 7-9, severe; and 10, most severe. Patients without intraoperative complications (pneumoperitoneum requiring needle drainage, severe arterial bleeding requiring the use of hemostatic forceps or severe mucosal injury) and severe pain (VAS score > 6) and nausea/vomiting at the fourth postoperative hour were given the early feeding approach. The severe mucosal injury was defined as the rupture of the mucosa above the tunnel. Enteral feeding was initiated in patients who did not match these requirements after 24h.

Patients were excluded from the study 1) if high-resolution manometry (HRM) failed (unable to tolerate HRM); 2) if the patient had undergone POEM for nonachalasia spastic esophageal motility disorders; 3) if the patient had gastric surgery; 4) if patient was younger than 18 years old; 5) if post-POEM follow-up was less than 3 months.

The study protocol was approved by the Ethics Committee of Ege University (approval date: March 23, 2023; approval no: 23-3.1T/14). Verbal and written informed consent was obtained from the patients who agreed to take part in the study.

### Procedural Details

Patients were administered nystatin drops 5 days before procedure. Simultaneously, patients had been fed fully and clearly in the last 5 days. Patients were admitted to the hospital on the morning of the procedure.

Procedures were performed under general anesthesia and in an operating room. All patients were administered 1 g of ceftriaxone intravenously as antibiotic prophylaxis 30 minutes before POEM. A single endoscopist (A.M.B.) performed all procedures. During procedures, a flush knife of 3 mm (Fujifilm, Tokyo, Japan) was used to open the submucosal tunnel, and a conventional triangle-tip knife (KD-640L, Olympus, Japan) was used in both esophageal and gastric myotomies. During the procedures, an ESG 400 Olympus cautery equipment (Olympus, Tokyo, Japan) was used. During mucosotomy, a pulse slow-cut effect of 2/40 watts was employed, and a spray coagulation mode (effect 2/30 watts) was used to open the submucosal tunnel. During myotomy, the pulse cut slow mode and spray coagulation mode were used alternately.

After myotomy, the lumen was washed with 80 mg of gentamicin diluted with 20 cc saline, and then, mucosectomy was closed with through-the-scope clips. As perioperative analgesics, 1 g of acetaminophen and 100 mg of tramadol were administered. The pain was assessed 4 and 24 hours post procedure. Second-look endoscopy was conducted 24 hours postoperatively in patients with a VAS score of >3 even after administering 3 g/day of acetaminophen and 100 mg/day of tramadol. No patients had a control esophagogram. Furthermore, patients’ chest x-rays and standard blood tests were performed on post POEM-day 1. Pneumothorax, pleural effusion, pneumonia, early mucosal detachment, and bleeding leading to prolongation of hospital stay were evaluated as severe complications.^9^

Intravenous antibiotic treatment with 2 g/day of ceftriaxone and 1.5 g/day of metronidazole was continued for 24 hours post POEM. For maintenance, patients were given a 3-day course of oral amoxicillin/clavulanic acid suspension (3 g/day). Furthermore, patients were advised to take 20 mg of rabeprazole per a day for the first 3 months. It was planned to follow a clear fluid diet for the first 3 days post POEM with soft meals for 10 days. After the second week, normal food was introduced.

### Data Collection

Patients’ demographic data, including age at diagnosis, sex, disease duration, body mass index (BMI), Charlson comorbidity index (CCI), symptom severity, and previous treatments were recorded. Endoscopic findings, HRM, and esophagography findings were recorded. Procedural details such as POEM time, lengths of esophageal and gastric myotomy were also recorded. The Eckardt score (ES) was evaluated to assess symptom severity at baseline.^9^ The treatment success was evaluated as an ES ≤3 at 3 months.

Patients were given a phone number to call if any problems occur post-discharge. Furthermore, the patient’s admission to the emergency department during the first 30 days of discharge was monitored using the health ministry’s electronic recording system. Patients’ reasons for admissions and examinations were investigated.

### Study End-points

1. The presence of complications associated with early feeding and the 30-day emergency readmission rate after POEM was the primary endpoints of the study.
2. Demographic data, achalasia subtypes, POEM procedure times, myotomy lengths, and length of hospital stay was secondary endpoints for comparing the early and late feeding groups.

### Statistical Analysis

The Kolmogorov–Smirnov test was used to analyze normally distributed numerical variables, which were given as mean ± standard deviation, and Student’s *t*-test was used for comparisons. Non-normally distributed numerical variables were expressed as median (interquartile range), and Mann–Whitney *U*-test was used for comparisons. Categorical variables were given as frequency (percentage), and comparisons were made using the chi-square test or Fisher’s exact test as appropriate. The chi-square test or Fisher’s exact test was performed to test the significance of pairwise differences using Bonferroni correction to adjust for multiple comparisons of achalasia types. The Statistical Package for the Social Sciences, Statistics for Windows, version 25.0 (IBM Corp., Armonk, NY, USA), was used for statistical analysis, and a *P*-value of <.05 was considered significant.

## Results

Between January 3, 2022, and December 25, 2022, a total of 121 patients were screened. Fourteen patients whose HRM was poorly tolerated, 3 patients who underwent POEM for nonachalasia spastic esophageal motility disorders (1 nutcracker, 1 diffuse esophageal spasm, 1 jackhammer esophagus), 2 patients with a history of bariatric surgery (sleeve gastrectomy), a patient younger than the age of 18, a patient with a follow-up was less than 3 months were excluded from the study. Finally, 100 patients were eligible for participation. Among these patients, there were 50 patients (50%) in the early feeding group, and 50 patients (50%) in the late feeding group. There were no significant differences between the 2 groups in terms of age, gender, BMI, CCI, symptom duration, and a previous history of POEM. Table 1 shows the characteristics of the whole study group and subgroups based on the enteral nutritional status of patients.

Dysphagia was the most prevalent primary symptom in both groups (n = 43, 86% in the early feeding group and n = 39, 78% in the late feeding group). The baseline symptom of chest pain was observed in 14 (28%) of patients in the early feeding group and 15 (30%) of patients in the late feeding group. Approximately one-third of the patients (n = 18, 36% in the early feeding group and n = 17, 34% in the late feeding group) had undergone previous therapy for achalasia, with PBD being the most prevalent treatment in treated patients (n = 15, 30% in the early feeding group and n = 14, 28% in the late feeding group). Five patients (n = 3, 6% in the early feeding group and n = 2, 4% in the late feeding group) had previously been treated with LHM. Type 2 achalasia was the most common achalasia subtype in both groups (n = 38, 76% vs. n = 27, 54%). Type 3 achalasia was more common in the late feeding group (n = 4, 8% vs. n = 12, 24%; *P* = .042). We found no statistically significant difference in duration of symptoms, initial ESS, HRM findings (integrated relaxation pressure, pressure of LES), esophageal width, endoscopic esophageal morphology. Procedural details (POEM time, length of submucosal tunnel or length of esophageal myotomy or length of gastric myotomy) were not significantly different between the 2 groups. Periprocedural complications occurred in 34 patients (68%) in the late feeding group, with pneumoperitoneum being the most common adverse event (n = 27, 54%). Table 2 lists the clinical characteristics, endoscopic findings, HRM findings, and procedure details of subgroups based on their enteral nutritional status.

In the early and late feeding groups, VAS scores were 4 (0-6) and 6 (1-8) (*P* < .001) at 4 hours and 1 (0-3) and 1 (0-6) (*P* = .043) at 24 hours, respectively. Severe pain occurred in 19 (38%) patients and nausea and/or vomiting occurred in 33 (66%) patients in the late feeding group at post-POEM fourth hour. No patients in the early feeding group required second-look endoscopy. Due to the persistent moderate-to-severe pain and suspected leakage, second-look endoscopy was performed on 5 patients (10%) in the late feeding group. While abnormal findings were observed in 3 patients (6%) after a second-look endoscopy, an intervention was indicated in a patient (2%) who developed massive pleural effusion. Second-look endoscopy showed that clips had fallen off the mucosal incision area. Three clips were applied for the closure of early mucosal detachment. Opiate use was statistically significantly higher in the late feeding group than in the early feeding group (n = 0, 0% vs. n = 18, 36%; *P* < .001). Only a patient in the early feeding group developed nausea and vomiting during follow-up. This patient was treated with parenteral antiemetic therapy. Post-POEM C-reactive protein (CRP) count values were lower in the early enteral feeding group vs. the late feeding group (CRP: median 27.24 gr/dL vs. 37.4 gr/dL; *P* = .037). We found no significant differences in post-POEM white blood cell or neutrophil count or hemoglobin values. Pathological findings on post-POEM chest x-ray were observed more frequently in the late-fed group. The median hospital stay in the early feeding group was 1 (1-3) days but 2 (1-4) days in the late feeding group, and this difference was statistically significant (*P* = .004). While no severe adverse event occurred in the early feeding group, unilateral pleural effusion causing prolonged hospitalization (4 days) was observed in a patient in the late feeding group. Although the 30-day emergent readmission rate was proportionally lower in the early feeding group vs. the late feeding group, no statistically significant difference was observed between the 2 groups (0% vs. 6%, respectively; *P* = .242). Other group comparisons revealed no statistically significant differences. Table 3 shows post-procedure outcomes of patients who comprised the entire study group as well as subgroups based on their feeding status.

## Discussion

Due to limitations in endoscopic procedures during the pandemic, Zhang et al^11^ discharged 14 out of 17 patients with severe symptomatic achalasia (ES ≥ 6) who met specific criteria (American Society of Anesthesiologists [ASA] I-III; no intraprocedural adverse events, responsive to medical treatment for postprocedural pain and nausea, and tolerating clear liquids) on the same day. The authors anticipated COVID-19 related indirect effects during post-POEM follow-up, but no problems were observed in the post-discharge follow-up of patients.^11^ Subsequently, a limited number of studies demonstrated no significant increase in morbidity after early feeding.^12-16^ This retrospective single-center study aimed to evaluate the effects of early feeding on patients who met specific criteria after POEM. The study included 100 patients, of whom 50% were started on early feeding. Only a patient in this group developed nausea and vomiting during follow-up. No significant increase in morbidity were observed with early feeding after POEM. The present study showed that early feeding in patients without complications and post-operative pain and nausea did not lead to serious complications.

The usefulness and necessity of control esophagography and second-look endoscopy before feeding in patients undergoing POEM are controversial. El Khoury et al^17^ examined the position of control esophagography after POEM. The sensitivity was 100%, but specificity was 45%. Control esophagography was not performed on any patient in our research because of its limited diagnostic value. Regarding the requirement for second-look upper endoscopy after POEM, the rate of abnormal findings in second-look endoscopy was 14.3%, whereas the rate of abnormal findings requiring therapy was 2.3% in Fujiyisho et al’s^18^ study. This study also showed that early enteral feeding without control esophagography and second-look endoscopy is safe in selected patients. Second-look endoscopy was recommended in cases of prolonged procedure time and intraoperative adverse events. Unlike other studies, here second-look endoscopy was conducted 24 hours postoperatively in patients with persistent pain. Although approximately 70% of patients in the delayed feeding group had perioperative complications, only 5 of them with persistent pain required a second-look endoscopy, and one of them had interventions. Based on this viewpoint, second-look endoscopy can be performed in patients experiencing particularly post-POEM persistent pain.

The optimal time of the patient’s discharge after POEM remains unknown. The average length of hospital stay in Inoue’s first series on the POEM approach was 4.8 days.^9^ Few recent studies highlight the same-day discharge following early feeding after POEM. Benias et al^13^ assessed the data of 103 patients who underwent POEM and were discharged on the same day and identified that postoperative chest pain and procedural difficulty were roadblocks to early discharge. Cloutier et al^14^ conducted a Canadian-based trial in which 72 (79.1%) of 90 POEM patients with pain responsive to medical treatment and who could tolerate liquid nutrition were discharged on the same day. Attaar et al^15^ who evaluated 115 patients who underwent POEM, reported that 48% of patients with nausea or pain responding to postoperative medical treatment with no preoperative complications were discharged on the same day. Considering the emergency department readmission rate of up to 12.7% reported in these trails,^11-16^ we believe that 1-day hospital observation may be an ideal strategy for post-POEM follow-up. In the present study, 42 patients (84%) in the early feeding group were discharged on the first postoperative day. Eight patients in the early feeding group were hospitalized for a longer time due to a return trip plan. There was no emergency department readmission in any of our patients after discharge. Therefore, the present study findings suggest that early feeding may be reliable in selected patients who meet certain criteria after POEM. Overnight hospital stay may reduce emergency readmission rates. However, further research is needed to support this recommendation. We currently advocate early feeding and discharge for patients without severe pain, nausea, or intraoperative complications, except for pneumoperitoneum that does not require drainage. In this way, health costs will be reduced as hospitalization decreases.

The study has some limitations that should be addressed. First, the sample size was small, and the study was conducted at a single center, which may limit the generalizability of findings. Second, the groups had a significant bias. As expected, the late feeding group had statistically significantly more periprocedural problems and higher 4- and 24-hour VAS scores, nausea, and vomiting rates. At this point, our study indicated that early feeding was directly a result of perioperative uncomplicated procedure and postoperative clinic. As a result of this, a statistically significant difference in hospitalization was discovered between the early- and late-feeding groups. The strongest aspect of our study is that it is the first study evaluating the early feeding in patients who underwent POEM during the hospital stay. Although the current study suggests that early feeding may be more cost-effective, no cost-effectiveness analysis was conducted. This subject should be carried out in the future. It is important to highlight that the categorization of early and late feeding groups was based on the occurrence of complications, pain, and gastrointestinal symptoms. The analysis considered both groups, whether complications were present or not. As a result, the findings are not unexpected and may not accurately represent the true “impact” of early nutrition. However, since what we want to highlight in our article is to create an algorithm for selecting patients who can be fed early after POEM, rather than the advantages of early feeding, we think that we have reached a conclusion on this issue as a result of our study.

In conclusion, our study showed that early feeding can be started at the fourth hour post procedure in patients who do not have severe pain, who do not develop nausea or vomiting unresponsive to treatment, and who do not have serious intraoperative complications. We speculate that the same criteria may also be useful in predicting conditions such as the need for second-look endoscopy, the need for opioids, and the need for prolonged hospitalization. We believe that using our approach, patients can be fed early and discharged early, thus reducing the length of hospital stay and health expenditures.

## Figures and Tables

**Table 1.** Patients’ Characteristics of the Study Group and Subgroups According to the Time to Feeding

	Early Feeding Group (n = 50)	Late Feeding Group (n = 50)	*P*
Age, mean, years	49.44 ± 14.7	50.46 ± 16.04	.741
Gender, male, n (%)	20 (40)	20 (40)	1.000
BMI (kg/m^2^)	24.98 ± 4.1	24.86 ± 4.29	.907
Smoking, n (%) Active smoker None Ex-smoker	12 (24) 34 (68) 4 (8)	15 (30) 28 (56) 7 (14)	.421
Alcohol consumption, n (%)	9 (18)	14 (28)	.235
Antiaggregant drug usage, n (%)	3 (6)	6 (12)	.487
CCI, median	1 (0-8)	1 (0-7)	.446

Results are expressed as mean ± standard deviation, median (interquartile range), or frequency (%). Significant *P*-values are in bold.

BMI, body mass index, CCI, Charlson comorbidity index.

**Table 2.** Clinical Data, Laboratory Variables, and Procedural Details of the Study Group and Subgroups According to Time to Feeding

	Early Feeding Group (n = 50)	Late Feeding Group (n = 50)	*P*
Initial symptom, n (%) Dysphagia (1) Chest pain (2) Regurgitation (3) (1) and (2) (1) and (3) (1) and (2) and (3)	30 (60) 1 (2) 5 (10) 2 (4) 3 (6) 8 (16)	30 (60) 7 (14) 3 (6) - 1 (2) 8 (16)	.153
Duration of symptoms, months	24 (3-300)	33 (3-240)	.097
Previous treatments, n (%) * *Treatment-experienced * *Naive	18 (36) 32 (64)	17 (34) 33 (66)	.834
Initial ESS	9 (5-12)	10 (6-12)	.245
IRP, median (mmHg)	24 (12-55.78)	26 (6-50)	.685
Pressure of LES, median (mmHg)	36.5 (10-80)	33 (16-78)	.290
Achalasia subtype, n (%) * *1 * *2 * *3	8 (16) 38 (76) 4 (8)	11 (22) 27 (54) 12 (24)	**.042**3
POEM time (min)	47.5 (30-120)	51 (28-115)	.588
Length submucosal tunnel (cm)	14 (10-21)	14 (8-27)	.487
Length of esophageal myotomy (cm)	7 (4-14)	6,5 (3-18)	.652
Length of gastric myotomy (cm)	3 (3-3)	3 (3-3)	1.000
Periprocedural complication, n (%) None Bleeding Pneumoperitoneum Severe mucosal injury Pneumoperitoneum and subcutaneous emphysema Pneumoperitoneum and bleeding	50 (100) - - - - -	16 (32) 2 (4) 27 (54) 1 (2) 2 (4) 2 (4)	**<.001**3

Results are expressed as mean ± standard deviation, median (interquartile range), or frequency (%). Significant *P*-values are in bold.

ESS, Eckardt symptom score; IRP, Integrated relaxation pressure; LES, lower esophageal sphincter; POEM, peroral endoscopic myotomy.

**Table 3.** Postprocedural Outcomes of the Study Group and Subgroups According to Time to Feeding

	Early Feeding Group (n = 50)	Late Feeding Group (n = 50)	*P*
Nausea or vomiting in the fourth hour, n (%)	-	33 (66)	**<.001**3
Fourth-hour VAS score > 6, n (%)	-	19 (38)	**<.001**3
24th-hour VAS score, median	1 (0-3)	1 (0-6)	**.043**3
Fever, n (%)	-	1 (2)	1.000
POD1 WBC count, median (10^9^/L)	10.33 (5.47-18.4)	10.45 (4.66-17.4)	.809
POD1 Neutrophil count, median (10^9^/L)	7.67 (3.5-15.87)	7.92 (3.35-16.34)	.994
POD1 Hemoglobin value, median (g/dL)	12.15 (2,.85-16)	12.15 (8.4-15.1)	.920
POD1 Platelet count, mean (10^9^/L)	219.14 ± 53.65	225.13 ± 50.86	.970
POD1 CRP, median	27.24 (3.3-92.77)	37.4 (9.1-128.65)	**.037**3
POD1 Chest x-ray (%) Normal Pneumoperitoneum (1) Pleural effusion (2) Pneumonia (3) (1) and (2) (2) and (3)	31 (62) 14 (28) 2 (4) 2 (4) 1 (2) -	16 (32) 26 (52) 3 (6) - 4 (8) 1 (2)	**.003**3
Nausea or vomiting after feeding	1 (2)	6 (12)	**.042**3
Second-look endoscopy, n (%)	-	5 (10)	.056
Post-POEM major adverse event	-	1 (2)	1.000
Opiate usage, n (%)	-	18 (36)	**<.001**3
Length of hospital stay, days	1 (1-3)	2 (1-4)	**.004**3
30-day emergent readmissions, n (%)	-	3 (6)	.242
First-month ESS, median	0 (0-4)	0 (0-3)	.142
Third-month ESS, median	0 (0-4)	0 (0-4)	.856
Clinical success, n (%)	49 (98)	49 (98)	1.000
Follow-up time, median, months	8 (3-15)	9 (3-15)	.838

Results are expressed as: median (interquartile range) or frequency (%). Significant *P*-values are in bold.

CRP, C-reactive protein; ESS, Eckardt symptom score; POD1, post-op day 1; VAS, visual analog scale; WBC, white blood cell.
